# Effects of exercise on serum ischemia-modified albumin, brain natriuretic peptide and copeptin levels in boxers and kick boxers

**DOI:** 10.11604/pamj.2022.41.98.23415

**Published:** 2022-02-03

**Authors:** Hikmet Memmedov, Ebubekir Bakan, Nurinnisa Ozturk, Nurcan Kılıc Baygutalp, Omer Kaynar, Mehmet Ali Gul, Abdulsamed Kaya, Fatih Kıyıcı, Ahmet Gokhan Yazıcı

**Affiliations:** 1 Azerbaijan Medical University, Faculty of Medicine, Department of Medical Biochemistry, Baku, Azerbaijan,; 2 Ataturk University, Faculty of Medicine, Department of Medical Biochemistry, Erzurum, Turkey,; 3 Department of Biochemistry, School of Pharmacy, Ataturk University, Erzurum, Turkey,; 4 Mus Alparslan University, Faculty of Education, Department of Physical Training and Sports, Mus, Turkey,; 5 Amasya University, Faculty of Medicine, Department of Medical Biochemistry, Amasya, Turkey,; 6 Mus Alparslan University, Vocational School of Health Services, Department of Medical Services and Techniques, Mus, Turkey,; 7 Ataturk University, Faculty of Sport Science, Erzurum, Turkey,; 8 Ataturk University, Faculty of Kazim Karabekir Education, Department of Physical Training and Sports, Erzurum, Turkey

**Keywords:** Brain injuries, traumatic, exercise therapy, natriuretic peptide, brain

## Abstract

**Introduction:**

boxing and kick boxing are combat sports that can cause severe head, neck, face and hand injuries during fighting. Then, traumatic brain injury (TBI) incidence is high in these sports. Ischemia-modified albumin (IMA), brain natriuretic peptide (BNP) and copeptin have diagnostic and prognostic value for cardiac and non-cardiac ischemic events. The purpose of this study is to evaluate exercise-induced variations of serum IMA, BNP and copeptin.

**Methods:**

twenty male boxers, twenty-three male kick boxers and twenty-three age-matched male were enrolled in the study. Health assessment data were analysed. Boxers and kick boxers underwent an exercise program including training plus fighting matches. Serum samples were collected in the pre- and post-exercise periods. Serum IMA, BNP and copeptin concentrations were measured in these specimens using ELISA reagents.

**Results:**

comparative analysis of analytes before and after exercise showed that exercise significantly increased serum IMA, BNP and copeptin levels both in boxers and kick boxers.

**Conclusion:**

in conclusion, IMA, BNP and copeptin levels may be candidate biomarkers for exercise-related traumatic brain injuries. The identification of new biomarkers in patients with acute and chronic neurological disorders is of considerable interest to clinicians. Then, further studies should be conducted to evaluate the possible role of IMA, BNP and copeptin in TBI pathophysiology.

## Introduction

There has been a growing interest in boxing, kick boxing and other martial arts due to the benefits of personal protection and keeping the body fit. Since boxing and kick boxing are combat sports, they are closely associated with health risks. Hitherto, various studies have been conducted to investigate traumatic effects of high-intensity exercise on some laboratory parameters including enzymes, lipids, some injury biomarkers, hormones, and others [1-5].

Traumatic brain injury can be defined as a concussion, impact or penetrating head injury that causes normal brain functions to deteriorate [6]. Professional boxers are at risk of acute and long-term neurological injury as the head area is the main target of the opponent [7]. Acute neurological injuries show a wide spectrum from mild concussion to cerebral hemorrhage, diffuse-axonal injury and death. Acute traumatic brain injury (ATBI) represents the neurologic consequence of concussive and sub concussive blows to the head. Evidence suggests that ATBI may be associated with boxing and collision sports such as American football and soccer, thus potentially exposing millions of athletes annually. Boxing is associated with acute neuronal and astroglial injury [8].

A review of the available records indicates that there have been a substantial number of fatalities in boxers due to intracranial injuries sustained in the ring in comparison to the numbers engaged at both amateur and professional levels [7]. The sport of boxing has been the source of much debate, with concerns about the neurological risks of participating having led to many calls to ban the sport [9]. Of various health risks of boxing and kick boxing, the most serious one is head injury related to repetitive blows on the head, neck, hands and face during fighting matches and training [10]. Chronic traumatic brain injury (TBI) has been reported in about 20% of professional boxers [11]. It is needed to determine concussion biomarkers providing diagnostic, prognostic, and monitoring information after injury. As a result of numerous studies conducted for this purpose, more than ten biomarkers of injury, including S100β, glial fibrillary acidic protein, neuron-specific enolase, tau, neurofilament light protein, amyloid beta, brain-derived neurotrophic factor, creatine kinase, heart-type fatty acid binding protein, prolactin, cortisol, and albumin, have been suggested to be concussion biomarkers [12]. Indeed, none of them is validated. Ischemia modified albumin (IMA) is a sensitive biomarker of acute ischemic events, particularly acute myocardial ischemia.

Few studies investigating the diagnostic role of IMA in non-cardiac ischemic states have suggested that elevated IMA levels may be considered as a sensitive biomarker of ischemic stroke [13]. Brain natriuretic peptide (BNP) is the N-terminal part of the pro-B type natriuretic peptide consisting of 32 amino acids released from cardiac ventricles in response to volume loads. Besides its substantial diagnostic and prognostic values in cardiovascular diseases, considerable data have shown that altered serum BNP levels have a prognostic value in non-cardiac acute ischemic events caused by head trauma [14]. Another sensitive biomarker, copeptin, is a stable peptide of the arginine vasopressin precursor and indicated to have diagnostic and prognostic values in a number of diseases including stroke and traumatic brain injury (TBI). High serum copeptin levels are also associated with the severity of injury and mortality due to TBI [15]. In this study, we aimed to evaluate the acute effects of high intensity exercise and trauma on serum levels of IMA, BNP and copeptin as biomarkers of cellular damage.

## Methods

**Study design and settings:** the research group was composed of 20 volunteer boxers from Atatürk University Kazim Karabekir Faculty of Education Department of Physical Education and Sports, 23 volunteer elite athletes (professional kickboxers) from Erzurum Pasinler Municipality Kickboxing sports club, and 23 volunteer participants who are healthy and have no sports background. The collected samples were studied in the Medical Biochemistry Department of Erzurum Atatürk University Medical Faculty Hospital in March 2014.

**Study population:** twenty healthy professional boxers (age range: from 16 to 32 years and BMI range: from 19.5 to 24.8; all male), 23 healthy professional kick-boxers (age range: from 17 to 32 years and BMI range: from 19.0 to 24.2; all male) and 23 healthy controls (age range: from 18 to 32 years and BMI range: from 19.1 to 24.6; all male) were included in the study. All participants were Caucasian. Individual information forms about sports and medical history of participants were fulfilled by an expert physician. The participants had no chronic disease and were not taking any medications. All sportsmen were regularly-training ones for at least three years at a frequency of at least three days per week. Boxers, kick-boxers and healthy control groups were consisted of participants with similar demographical characters, showing homogenous groups (Table 1).

**Table 1:** demographical characteristics of the 20 healthy professional boxers, 23 healthy professional kick-boxers and 23 healthy controls included in the study

Demographical characteristics	Boxers	Kick-boxers	Controls
**Age (years, mean ± SD)**	20.21 ± 3.35^a*^	20.08 ± 3.6^b*^	21.04 ± 3.82
**Body weight (kg)**	69.82 ± 9.39^a*^	65.20 ± 10.41^b*^	65.18 ± 10.51
**BMI**	22.52 ± 2.12^a*^	22.11 ± 3,04^b*^	22.15 ± 3.26
**Duration of sports (years, mean ± SD)**	3.8 ± 1.7	4.0 ± 1.8	-
**No of male/female**	20/0	23/0	23/0

(BMI: Body mass index, comparisons ^a^boxing sportsmen vs healthy controls, ^b^kick-boxers vs healthy controls, (*): p>0.05, Student's t test)

**Exercise planning and sampling:** the physical activity consisted of boxing and kick-boxing matches of five minutes following a training for 25 minutes, and the combination of the two activities was called as exercise. Exercise program was practiced in the morning hours. Venous blood samples were taken from sportsmen twice: first sampling before exercise and second sampling immediately after exercise. Blood samples from the athletes were taken by Dr. Nurinnisa Öztürk, Dr. Mehmet Ali Gül and Dr. Hikmet Mammadov collected it and kept it in suitable conditions for research.

**Data collection:** serum IMA, BNP and copeptin levels were measured by ELISA with commercial kits (Cusabio; China, Raybio; USA, Cloud-Clone Corp; USA, respectively) according to the manufacturer´s instructions. Adrenocorticotropic hormone (ACTH) and cortisol were analyzed by chemiluminescence method in Siemens Immulite 2000 analyzer and Beckman Coulter DXI800 analyzer, respectively. Serum creatinine measurements were performed by kinetic modification of the Jaffe procedure on Beckman Coulter AU5800 (Beckman Coulter Inc., Brea CA, USA). To evaluate the kidney functions of sportsmen, estimated glomerular filtration rates (EGFR) were calculated using the formula described by Levey *et al*. [16].

**Statistical analysis:** data were analyzed using the SPSS statistical software package (SPSS, v.20.0 for Windows, SPSS Inc. Chicago). All results were expressed as mean ± standard deviation. Normality of biochemical parameters was analyzed by Kolmogorov-Smirnov test. Kolmogorov-Smirnov test was used to reveal whether the data were suitable for normal distribution. Analyze - Nonparametric Tests - 1-Sample K-S process was followed, the variables to be tested for normality were transferred to the “Test Variable List” cell in the opened window, and the “Output” file created was evaluated for the details of the analysis. Since all parameters were normally distributed the Student´s t test and paired samples t-tests were used to evaluate the significance of differences between groups (Student´s t test for: controls values vs pre-exercise values of boxers and control values vs pre-exercise values of kick-boxers; paired samples t-test for: pre-exercise values of boxers vs post-exercise values of boxers and pre-exercise values of kick-boxers vs post-exercise values of kick-boxers). Pearson correlation analysis were used. P values less than 0.05 were considered significant, at 95% confidence interval. Post-exercise values were adjusted due to do decrement in the plasma volume using a formula suggested by Dill and Costill [17].

**Ethical statement:** the study protocol was reviewed and approved by an institutional ethical board (26.12.2013/6). Informed consent was obtained from each participant and patient anonymity was preserved. Study protocol was in accordance with the Helsinki Declaration of 1975, as revised in 1983.

## Results

Demographical data of sportsmen are given in Table 1. There were no significant differences in terms of body weight and body mass index (BMI) values of boxers compared to healthy controls, and kick-boxers compared to healthy controls (p>0.05).

Body weights of boxers significantly decreased from 69.82 ± 9.39 kg to 69.25 ± 9.48 kg and BMI values significantly decreased from 22.52 ± 2.12 to 22.33 ± 2.16 by exercise (p<0.001 for both parameters). These changes accounted for approximately 1% decrement in plasma volumes of boxers. Similarly, body weights of kick-boxers significantly decreased from 65.20 ± 10.41 kg to 62.08 ± 16.87 kg and BMI values significantly decreased from 22.11 ± 3.04 to 22.01 ± 3.05 by exercise (p<0.001 for both parameters). These changes accounted for approximately 6% decrement in plasma volumes of kick-boxers.

Statistical evaluations of the biochemical analytes are given in Table 2, Table 3, Table 4. Pre-exercise levels of IMA, BNP and copeptin levels in boxers and kick-boxers were compared to healthy controls. There were no significant differences in pre-exercise IMA, BNP and copeptin levels of boxers and kick-boxers compared to the healthy controls (boxers vs healthy controls: p>0.05 for all parameters; kick-boxers vs healthy controls: p>0.05 for all parameters) (Table 2).

**Table 2:** evaluation of pre-exercise IMA, BNP and Copeptin levels in 20 healthy professional boxers, 23 healthy professional kick-boxers and 23 healthy controls included in the study

Analytes	Boxers (n=23)	Kick-boxers (n=23)	Controls (n=23)
**IMA (pg/ml)**	13,95 ± 10,06 ^a*^	12,00 ± 6,47^b*^	11,87 ± 8,33
**BNP (pg/ml)**	4,93 ± 2,33^a*^	6,52 ± 1,63^b*^	4,84 ± 0,72
**Copeptin (pg/ml)**	4.38 ± 1.04^a*^	3,81 ± 1,27^b*^	3,54 ± 0,71

(IMA: ischemia-modified albumin, BNP: brain-type natriuretic peptide, Comparisons. ^a^boxers vs healthy controls; ^b^kick-boxers vs healthy controls; ^*^p>0.05, Student's t test)

**Table 3:** evaluation of some biochemical parameters in 20 healthy professional boxers included in the study, before and after exercise

Analytes	Pre-exercise	Post-exercise 1	Post-exercise 2	p value
**IMA (pg/ml)**	13.95 ± 10.06	23.35 ± 9.56	23.11 ± 9.46	<0.001
**BNP (pg/ml)**	4.93 ± 2.33	6.87 ± 1.31	6.80 ± 1.30	<0.001
**Copeptin (pg/ml)**	4.38 ± 1.04	60.67 ± 28.35	60.06 ± 28.06	<0.001
**Creatinine (mg/dl)**	0.94 ± 0.13	1.10 ± 0.15	1.09 ± 0.14	<0.001
**EGFR**	100.19 ± 15.72	83.25 ± 14.32	82.42 ± 14.18	<0.001
**ACTH (pg/mL)**	18.41 ± 8.71	31.12 ± 15.41	30.81 ± 15.25	<0.001
**Cortisol (pg/ml)**	9.27 ± 3.25	9.81 ± 2.54	9.71 ± 15.10	0.070

(Results are shown as mean ± SD; BMI: Body mass index, IMA: ischemia-modified albumin, BNP: brain-type natriuretic peptide, EGFR: estimated glomerular filtration rate, ACTH: adrenocorticotropic hormone, Post-exercise 1: measured post-exercise values, Post-exercise 2: adjusted post-exercise values according to the plasma volume changes, p value: significance value for comparison of pre-exercise vs post-exercise-2 (paired samples t-test).

**Table 4:** evaluation of some biochemical parameters in 23 healthy professional kick-boxers included in the study, before and after exercise

Analytes	Pre-exercise	Post-exercise-1	Post-exercise 2	p value
**IMA (pg/ml)**	12.00 ± 6.47	20.95 ± 8.23	19.70 ± 7.74	<0.001
**BNP (pg/ml)**	6.52 ± 1.63	8.34 ± 1.86	7.84 ± 1.75	<0.001
**Copeptin (pg/ml)**	3.81 ± 1.27	50.40 ± 33.74	47.38 ± 31.71	<0.001
**Creatinine(mg/dl)**	0.96 ± 0.14	1.11 ± 0.15	1.04 ± 0.14	<0.001
**EGFR**	97.20 ± 27.74	81.94 ± 23.44	77.02 ± 22.03	<0.001
**ACTH (pg/mL)**	17.66 ± 7.97	52.58 ± 38.51	49.42 ± 36.20	<0.001
**Cortisol (pg/ml)**	10.48 ± 2.86	11.10 ± 2.87	10.43 ± 2.70	0.075

(Results are shown as mean ± SD; BMI: Body mass index, IMA: ischemia-modified albumin, BNP: brain-type natriuretic peptide, EGFR: estimated glomerular filtration rate, ACTH: adrenocorticotropic hormone, Post-exercise 1: measured post-exercise values, Post-exercise 2: adjusted post-exercise values according to the plasma volume changes, p value: significance value for comparison of pre-exercise vs post-exercise-2 (paired samples t-test).

**Figure 1:**
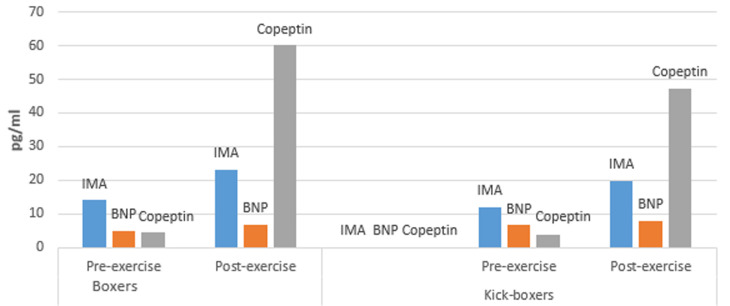
pre-exercise and post-exercise serum IMA, BNP and Copeptin levels in boxers and kick-boxers

There were significant differences between pre- and post-exercise levels of IMA, BNP, copeptin and ACTH both in boxers and kick-boxers (p<0.05 for each parameter, Table 3, Table 4, Figure 1). On the other hand, there was no significant difference between pre- and post-exercise levels of cortisol in boxers and kick-boxers (p>0.05 for both groups, Table 3, Table 4).

When Pearson correlation analyses were performed to evaluate relationships between biochemical values of sportsmen, a significant correlation was found only in post-exercise BNP and copeptin values of kick-boxers (p=0.006, r=0.553). The study was presented as a poster (Published as Poster Abstracts) at the 21^st^ IFCC-EFLM European Congress of Clinical Chemistry and Laboratory Medicine at Paris.

## Discussion

There are a few studies investigating the variations of IMA, BNP and copeptin after exercise [18, 19]. In this study we aimed to analyze the effects of short-term and intense exercise on serum IMA, BNP and copeptin levels in boxers and kick-boxers.

Our results showed that there were significant differences between pre- and post-exercise levels of IMA, BNP, copeptin and ACTH both in boxers and kick-boxers. In order to prevent the confounding effects of the decline in the plasma volume caused by weight loss, we performed an adjustment suggested by Dill and Costill [17]. When we re-evaluated the effects of exercise on biochemical parameters considering the plasma volume changes about 1% in boxers and about 6% in kick-boxers, we determined that post-exercise values were still significantly higher than pre-exercise values of IMA, BNP and copeptin both in boxers and kick-boxers. Besides, we evaluated the pre-exercise kidney functions of sportsmen according to the calculation of estimated glomerular filtration rate (EGFR) by using serum creatinine values. Since the calculated EGFR values were higher than 90, kidney functions of sportsmen were accepted normally. Also, in order to eliminate the probability that copeptin values might be altered because of the stress occurred in the fighting matches, we analyzed the serum cortisol and ACTH values both before and after exercise. Results showed that there were no significant differences in terms of pre-exercise and post-exercise levels of cortisol both in boxers and kick-boxers. Although serum ACTH values were significantly altered by exercise both in boxers and kick-boxers, the post-exercise values of ACTH in boxers were within the reference ranges we have been using in our laboratory and the post-exercise values of ACTH in kick-boxers were nearby the upper limit of reference range, which is 46 pg/ml for our laboratory.

Based on these results, we can conclude that although copeptin is reported to be altered in response to stress [18], the alteration in our study was arisen from traumatic injury rather than stress. Occurrences of severe injuries during trainings and fights have been reported in combat sports, particularly in the most contact ones, boxing and kick-boxing [10]. Chronic neurological symptoms resulted in exposure to repetitive concussions in boxers were previously entitled as the “punch drunk syndrome” [20]. The definition of “TBI” is replaced to before-mentioned old definition [21]. TBI has two types as acute and chronic [22]. Boxers and kick-boxers are at risk of acute and chronic traumatic brain injury due to acute and prolonged deleterious effects of high intensity exercise and blows. It is necessary to determine concussion biomarkers providing diagnostic, prognostic, and monitoring information after injury. The identification of specific and sensitive biomarkers of TBI is a widely-investigated subject. The present study tried to investigate the possible diagnostic values of BNP, IMA and copeptin in TBI.

IMA is a structurally different form of albumin and is produced by reactive oxygen species during ischemia. IMA has been referred to as a sensitive biomarker of myocardial ischemia [23, 24]. Further, there are several studies reporting high IMA values in non-cardiac ischemic events [13], and there are limited data suggesting serum IMA levels being a non-specific biomarker of tissue ischemia [25]. In our study, post-exercise serum IMA levels were significantly higher than pre-exercise ones both in boxers and kick-boxers. Our findings show that any form of ischemia existed in sportsmen, which is probably cardiac ischemia, due to the knowledge that whereas post-exercise increases in serum IMA levels within 24-48 hours is attributed to acute skeletal muscle ischemia, immediate increases may be attributed to cardiac ischemia [26]. It is important to take into consideration the fact that the susceptibility of cells to ischemia in different organs is variable and that the optimal IMA cut-off levels showing various ischemic events should be determined for different organs [13].

Natriuretic peptides, on the other hands, are a group of hormones with vasodilator and antiproliferative properties, which are produced against the vasoconstrictor effects of neurohormones released from sympathetic nerve system and renin-angiotensin aldosteron system. These peptides are produced in order to make natriuresis and diuresis via the increase in intracellular cGMP levels, and it is well known that the plasma natriuretic peptides rise in the hearth failure [27]. Consequently, they are considered as validated biomarkers of heart failure and used for the early diagnosis or elimination of heart failure [28]. Thus, the 2012 European Society of Cardiology guidelines report specific age-independent decision cut-off limits of natriuretic peptides for exclusion of heart failure [29].

Based on the usage of brain-type natriuretic peptide (BNP) in estimating stretch-activated heart failure, the diagnostic value of BNP in other stretch-activated conditions, such as head injury, is investigated [14, 30]. In a study conducted on patients with subarachnoid hemorrhage, plasma BNP levels were found to be significantly higher than those of controls [31]. Suppression of aldosterone synthesis following increased BNP secretion is proposed to result in hyponatremia in patients with subarachnoid hemorrhage. BNP is suspected to have role in TBI pathophysiology, since elevated serum levels of BNP is reported following head injury [32].

Copeptin, another parameter in this study, is the C-terminal part of arginine vasopressin precursor peptide (pro-AVP) and is a specific indicator of arginine vasopressin (AVP) release. The triggered co-release of AVP and copeptin in response to a stress stimulant from posterior pituitary results in ACTH and cortisol release [33]. Since altered copeptin levels were determined in many emergent clinical situations, copeptin is suggested to be a valuable prognostic biomarker in those situations including pulmonary diseases, heart disease, stroke, shock and traumatic brain injury. In a study conducted on 94 patients with acute severe TBI, copeptin was reported to be related to the severity of injury and mortality of TBI [15].

Cortisol have a tendency to increase as stress factor within 30-60 minutes following exercise [34]. In our study whereas ACTH levels were significantly altered by exercise, there were no significant differences in terms of cortisol levels in boxers and kick-boxers who were exposed to a total of 30 minutes of exercise including training and matching. These results may be due to the short duration of total exercise (30 minutes), which has not been sufficient to induce stress response.

## Conclusion

In conclusion, although sports is globally accepted as having numerous benefits to the body, it should be noted that it may not always be safe, especially in branches including exhaustive exercise and trauma. To the best of our knowledge, this is the first study underlining the possible importance of BNP, IMA and copeptin in boxers and kick boxers exposed to TBI. Since boxers and kick-boxers are highly exposed to acute and chronic brain injury, further studies to be conducted on this population including using of neuroimaging techniques may be helpful in identifying and clinical usage of new biomarkers for acute and chronic traumatic brain injury. Our results were in accordance with previous studies, showing altered levels of BNP, copeptin, ischemia modified albumin and ACTH after exercise in sportsmen exposed to head trauma. We suggest that sportsmen under high risk of head trauma be subjected to regular health controls including evaluation of BNP, IMA and copeptin, candidate biomarkers of traumatic brain injury.

### What is known about this topic

- Since boxing and kick-boxing are combat sports, serious injuries may occur to the head, neck, face and hands during fighting, and traumatic brain injury (TBI) incidence is high in these sports;
- Ischemia-modified albumin (IMA), brain-type natriuretic peptide (BNP) and copeptin have diagnostic and prognostic value in cardiac and non-cardiac ischemic events.

### What this study adds

- The comparative analysis of analytes before and after exercise showed that the exercise causes significant increase in serum IMA, BNP and copeptin levels both in boxers and kick-boxers;
- IMA, BNP and copeptin levels may be candidate biomarkers of exercise-related traumatic brain injury.

## References

[1] Khanna GL, Manna I (2006). Study of physiological profile of Indian boxers. J Sports Sci Med.

[2] Chatterjee P, Banerjee AK, Majumdar P, Chatterjee P (2007). Study of plasma lipid and lipoprotein profile in elite women boxers during a six weeks' training programme. JNMA J Nepal Med Assoc.

[3] Karakukcu C, Polat Y, Torun YA, Pac AK (2013). The effects of acute and regular exercise on calcium, phosphorus and trace elements in young amateur boxers. Clin Lab.

[4] Jordan BD (2000). Chronic traumatic injury associated with boxing. Semin Neurol.

[5] Tanriverdi F, Unluhizarci K, Kocyigit I, Tuna IS, Karaca Z, Durak AC (2008). Brief communication: pituitary volume and function in competing and retired male boxers. Ann Intern Med.

[6] Önal MB, Narin F, Berker M, Palaoğlu ÖS (2013). Sports related brain injury. Turkiye klinikleri journal of medical sciences.

[7] Ryan AJ (1987). Intracranial injuries resulting from boxing: a review (1918-1985). Clin Sports Med.

[8] Matser EJ, Kessels AG, Lezak MD, Troost J, Jordan BD (2000). Acute traumatic brain injury in amateur boxing. Phys Sportsmed.

[9] Paul McC, Tsharni Z, Peter C (2007). The evidence for chronic traumatic encephalopathy in boxing. Sports Med.

[10] Cynarski WJ, Kudłacz M (2008). Injuries in martial arts and combat sports-a comparative study. Archives of Budo.

[11] Jordan BD (2000). Chronic traumatic brain injury associated with boxing. Semin Neurol.

[12] Papa L, Ramia MM, Edwards D, Johnson BD, Slobounov SM (2015). Systematic Review of Clinical Studies Examining Biomarkers of Brain Injury in Athletes after Sports-Related Concussion. J Neurotrauma.

[13] Talwalkar SS, Bon Homme M, Miller JJ, Elin RJ (2008). Ischemia modified albumin, a marker of acute ischemic events: a pilot study. Ann Clin Lab Sci.

[14] Sviri GE, Soustiel JF, Zaaroor M (2006). Alteration in brain natriuretic peptide (BNP) plasma concentration following severe traumatic brain injury. Acta Neurochir (Wien).

[15] Dong XQ, Huang M, Yang SB, Yu WH, Zhang ZY (2011). Copeptin is associated with mortality in patients with traumatic brain injury. J Trauma.

[16] Levey AS, Coresh J, Greene T, Jane Marsh, Stevens LA, Kusek JW, Lente FV (2007). Expressing the Modification of Diet in Renal Disease Study equation for estimating glomerular filtration rate with standardized serum creatinine values. Clin Chem.

[17] Dill DB, Costill DL (1974). Calculation of percentage changes in volumes of blood, plasma, and red cells in dehydration. J Appl Physiol.

[18] Lippi G, Schena F, Salvagno GL, Sanchis-Gomar F, Guidi GC (2015). Serum copeptin and midregion proadrenomedullin (MR-proADM) after an ultramarathon. J Clin Lab Anal.

[19] Scharhag J, George K, Shave R, Urhausen A, Kindermann W (2008). Exercise-associated increases in cardiac biomarkers. Med Sci Sports Exerc.

[20] Martland HS (1928). Punch drunk. JAMA.

[21] Critchley E (1937). Nervous disorders in boxers. Medical Annual.

[22] Blennow K, Hardy J, Zetterberg H (2012). The neuropathology and neurobiology of traumatic brain injury. Neuron.

[23] Bar-Or D, Winkler JV, Vanbenthuysen K, Harris L, Lau E, Hetzel FW (2001). Reduced albumin-cobalt binding with transient myocardial ischemia after elective percutaneous transluminal coronary angioplasty: a preliminary comparison to creatine kinase-MB, myoglobin and troponin I. Am Heart J.

[24] Sinha MK, Gaze DC, Tippins JR, Collinson PO, Kaski JC (2003). Ischemia modified albumin is a sensitive marker of myocardial ischemia after percutaneous coronary intervention. Circulation.

[25] Sbarouni E, Georgiadou P, Voudris V (2011). Ischemia modified albumin changes-review and clinical implications. Clin Chem Lab Med.

[26] Apple FS, Quist HE, Otto AP, Mathews WE, Murakami MM (2002). Release characteristics of cardiac biomarkers and ischemia-modified albumin as measured by the albumin cobalt-binding test after a marathon race. Clin Chem.

[27] de Lemos JA, McGuire DK, Drazner MH (2003). B-type natriuretic peptide in cardiovascular disease. Lancet.

[28] Loncar G, Omersa D, Cvetinovic N, Arandjelovic A, Lainscak M (2014). Emerging biomarkers in heart failure and cardiac cachexia. Int J Mol Sci.

[29] Thygesen K, Mair J, Mueller C, Huber K, Weber M, Plebani M (2012). Recommendations for the use of natriuretic peptides in acute cardiac care: a position statement from the Study Group on Biomarkers in Cardiology of the ESC Working Group on Acute Cardiac Care. Eur Heart J.

[30] Lu DC, Binder DK, Chien B, Maisel A, Manley GT (2008). Cerebral salt wasting and elevated brain natriuretic peptide levels after traumatic brain injury: 2 case reports. Surg Neurol.

[31] Berendes E, Walter M, Cullen P, Prien T, Aken HV, Horsthemke J (1997). Secretion of brain natriuretic peptide in patients with aneurysmal subarachnoid haemorrhage. Lancet.

[32] Wu X, Sha H, Sun Y, Gao L, Liu H, Yuan Q (2011). N-terminal pro-B-type natriuretic peptide in patients with isolated traumatic brain injury: a prospective cohort study. J Trauma.

[33] Nickel CH, Bingisser R, Morgenthaler NG (2012). The role of copeptin as a diagnostic and prognostic biomarker for risk stratification in the emergency department. BMC Med.

[34] Frayn KN (2009). Metabolic regulation. A human perspective. Coping with some extreme situations.

